# Ventilation before Umbilical Cord Clamping Improves Physiological Transition at Birth or “Umbilical Cord Clamping before Ventilation is Established Destabilizes Physiological Transition at Birth”

**DOI:** 10.3389/fped.2015.00029

**Published:** 2015-04-20

**Authors:** David J. R. Hutchon

**Affiliations:** ^1^Obstetrics, Darlington Memorial Hospital, Darlington, UK

**Keywords:** neonatal, preterm birth, transition, umbilical cord clamping, delayed cord clamping, resuscitation with cord intact, intraventricular haemorrhage

Bhatt et al’s paper provides a good argument for ensuring that ventilation is established before clamping the umbilical cord (1). The possibility that very early clamping could explain the increased occurrence of intraventricular hemorrhage was proposed in 1988 by Hofmeyr (2) who showed that in human babies there was a marked rise in arterial pressure when the cord was clamped as late as 35 s after birth. He proposed that the sudden rise in cerebral circulation was the underlying mechanism for the higher incidence of intraventricular hemorrhage after early cord clamping (ECC) in preterm neonates. Our computer simulation of transitional circulation supports Hofmeyr’s finding (3). The simulation demonstrates that a sudden rise in cerebral pressure and blood flow is inevitable if the placental circulation is closed before the increase in pulmonary blood flow has occurred as a result of respiration. In the experimental lamb, Bhatt et al. have now provided further evidence of this adverse effect of early clamping (4). The instability in the circulation shown in their lambs may also be the result of loss of the blood volume (5) known to be redistributed from the placenta to the neonate (placental transfusion) and from the loss of the oxygenated blood from the umbilical vein. Long-term harms for the neonate may also result from loss of the stem cells capable of quickly repairing vital organ injury.

Charles White an eminent obstetrician in Manchester, England (6) could not have put it better in 1773

… the common method of tying and cutting the umbilical cord in the instant the child is born, is likewise one of those errors in practice that has nothing to plead in its favour but custom. Is it possible that this wonderful alteration in the human machine should properly be brought about in one instant of time, and at the will of a bystander? Let us leave the affair to nature, and watch her operations and it will soon appear that she stands not in need of our feeble assistance, but will do the work herself at a proper time, and in better manner. In a few minutes the lungs will gradually be expanded and the great alterations in the heart and blood vessels will take place. As soon as this is perfectly done, the circulation in the umbilical cord will cease itself… By this rash, inconsiderate method of tying the umbilical cord before the circulation in it is stopped, I doubt not but many children have been lost, many of their principal organs have been injured, and foundations laid for various disorders.

The practice of ECC has become so established it is often considered the physiological norm. The title “Ventilation before umbilical cord clamping improves the physiological transition at birth” concurs with this view. A statement such as “Umbilical cord clamping before ventilation is established destabilizes physiological transition at birth” focuses attention on the physiological starting point. While there has been debate about the timing of cord clamping for over 2000 years, it is only in recent times that the cord could have been routinely clamped within seconds of birth. One hundred fifteen years ago, the first umbilical cord clamp was devised by Magennis (7). He specifically advised practitioners to wait until the function of the cord had ceased before applying the clamp.

For those babies, who failed to breathe within a short interval, routine ventilation was only introduced quite recently (8). Although ECC by obstetricians may have already been common, most births were still at home. However, the finding in 1960s that post-partum hemorrhage (PPH), a major cause of maternal death, could be reduced by active management of the third stage of labor (AMTL), established ECC into obstetrical and hospital midwifery practice (9). With the majority of births now in hospital, AMTL became very common. ECC was included as one of the essential elements of active management included without any evidence of efficacy. The established practice of ECC as part of AMTL made neonatal research difficult. Since 1960s, studies have shown that ECC has no role in reducing maternal hemorrhage but ECC was by now recommended worldwide as good routine obstetric practice. The trials of DCC vs. ECC had small numbers and measured only short-term outcomes, so the realization by clinicians that ECC was an unnecessary and potentially harmful intervention for the neonate was very slow. Of significance was the exclusion of all neonates requiring resuscitation from the randomized controlled trials, and this was the very group most likely to show serious harms.

Concern was initially voiced by the few midwives who cared for women still birthing at home, and followed by a few neonatologists and obstetricians who recognized the importance of avoiding sudden interruption of the placental circulation. The first neonatologist to recognize the importance in recent times was Professor Peter Dunn. Working in several hospitals in England between 1961 and 1971, he commenced resuscitation of preterm infants as they lay on their mother’s legs before the cord had been clamped or the placenta delivered. This resulted in more than a threefold fall in perinatal mortality of these preterm neonates (6). This was well before the era of antenatal steroids.

One minute is usually long enough for the healthy term neonate to establish respiration and so for the majority simply waiting is all that is required. For the preterm or asphyxiated term neonate, the most effective way of initiating ventilation before clamping off the placental circulation has still to be established. It may not be so simple to achieve in practice without design and preparation. Modern ventilation requires pressure and volume to be carefully controlled to avoid injury to the lungs and ensuring the equipment is portable enough to reach the side of the mother is the challenge. Intubation may often be required, and hypothermia needs to be prevented. Thus, a more specialized approach is necessary, which is being met by a modified mobile neonatal resuscitation trolley (10). Clinical trials are already underway.

## Conflict of Interest Statement

I have no financial or gain in the production of the LifeStart trolley.All intellectual property has been handed over to Indithermplc in exchange for an obligation by Inditherm to make a donation to the neonatal charity BLISS for each trolley sold.
